# Development of a highly polymorphic chloroplast SSR set in *Abies grandis* with transferability to other conifer species—A promising toolkit for gene flow investigations

**DOI:** 10.1002/ece3.11593

**Published:** 2024-06-20

**Authors:** Jeremias Götz, Ludger Leinemann, Oliver Gailing, André Hardtke, Oliver Caré

**Affiliations:** ^1^ ISOGEN GmbH & Co KG Göttingen Germany; ^2^ Buesgen Institute, Forest Genetics and Forest Tree Breeding, Faculty of Forest Sciences and Forest Ecology University of Göttingen Göttingen Germany; ^3^ Department of Forest Genetic Resources, Breeding and Testing of Forest Reproductive Material Northwest German Forest Research Institute (NW‐FVA) Hann. Münden Germany

**Keywords:** *Abies*, chloroplast, conifer, grand fir, microsatellite, SSR

## Abstract

The genus *Abies* is widely distributed across the world and is of high importance for forestry. Since chloroplasts are usually uniparentally inherited, they are an important tool for specific scientific issues like gene flow, parentage, migration and, in general, evolutionary analysis. Established genetic markers for organelles in conifers are rather limited to RFLP markers, which are more labour and time intensive, compared with SSR markers. Using QUIAGEN CLC Workbench 23.03, we aligned two chloroplast genomes from different *Abies* species (NCBI accessions: NC_039581, NC_042778, NC_039582, NC_042410, NC_035067, NC_062889, NC_042775, NC_057314, NC_041464, MH706706, MH047653 and MH510244) to identify potential SSR candidates. Further selection and development of forward and reverse primers was performed using the NCBI Primer Blast Server application. In this article, we introduce a remarkably polymorphic SSR marker set for various *Abies* species, which can be useful for other conifer genera, such as C*edrus*, *Pinus*, *Pseudotsuga* or *Picea*. In total, 17 cpSSRs showed reliable amplification and polymorphisms in *A. grandis* with a total of 68 haplotypes detected. All 17 cpSSRs amplified in the tested *Abies* spp. In the other tested species, except for *Taxus baccata*, at least one primer was polymorphic.

## Graphical abstract

We introduce a remarkably polymorphic SSR marker set for various Abies species, which can be useful for other conifer genera, such as Cedrus, Pinus, Pseudotsuga or Picea. In total, 17 cpSSRs showed reliable amplification and polymorphisms in *Abies grandis* with a total of 68 haplotypes detected. All 17 cpSSRs amplified in the tested Abies spp. In the other tested species, except for Taxus baccata, at least one primer was polymorphic.

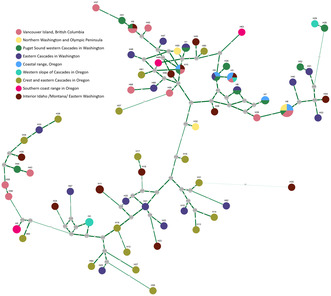

## 1 INTRODUCTION

Organelle genomes are commonly known to be inherited in various pathways in plants. Depending on the species, both, chloroplasts and mitochondria, are either inherited maternally, paternally or even by both parents together. Even within conifers, reports about different inheritance pathways are published (Adams, 2019), which is why comprehensive knowledge about the target species is obligatory for genetic studies in conifers. For *Abies* spp. and *Pinaceae*, in general, inheritance of chloroplasts usually follows the paternal line and mitochondria the maternal linen (Isoda et al., 2000; Vendramin & Ziegenhagen, 1997; Ziegenhagen et al., 1995). However, all conifer organelle genomes have in common, that their genomes´ silent mutation rates are much lower compared to nuclear sequences (Drouin et al., 2008; Wolfe et al., 1987). Hence, they tend to be more conservative compared with nuclear markers. This combined with the inheritance pathway makes genetic markers in organelles well suitable for gene flow, parentage, migration and, in general, evolutionary analysis. Established conifer chloroplast markers often use restriction enzymes to detect different alleles. However, such RFLP markers tend to be more labour intensive and less polymorphic compared to SSR and SNP markers (Powell et al., 1996). In this publication, we aligned 12 chloroplast genomes of nine different *Abies* species to detect regions of variation within the genus *Abies*. Subsequently, we designed and tested chloroplast SSR primer pairs for promising loci aiming for a polymorphic cpSSR set in *Abies grandis*. Primer candidates were tested in an *A. grandis* test sample set, which covers the complete native range of the species, as well as additional conifer species. In the present article, we introduce a highly polymorphic, easy–to‐use cpSSR marker set for *A. grandis* and additional conifer species.

## 2 MATERIALS AND METHODS

### 2.1 SSR identification and primer design

We aligned 12 chloroplast genomes of different *Abies* species (Table 1) using the QIAGEN CLC Workbench 23.03 (Qiagen, Hilden, Germany) to detect chloroplast DNA variation in the *Abies* genus. All sequences originate from the NCBI database (Sayers et al., 2021) in FASTA format. Manual browsing for regions of variation in short repetitive motives in the alignment revealed potential SSR candidates, of which we chose only those, flanked by conservative nucleotide sequences. The NCBI Primer blast server application (Ye et al., 2012) was used to develop forward and reverse primers in highly conservative strand regions around the SSR candidates. Position in the chloroplast genome (Table A1) of the selected (Table 2) polymorphic and the nonpolymorphic rejected (Table A2) markers was determined by blasting against the NCBI database.

**TABLE 1:** NCBI accession code and according to publication for aligned chloroplast genome sequences from different *Abies* species.

Species	Origin	Accession	Publication
*A. concolor*	North America	NC_039581	Gernandt et al. (2018)
*A. balsamea*	North America	NC_042778	Wu et al. (2019)
*A. religiosa*	Central America	NC_039582	Gernandt et al. (2018)
*A. alba*	Europe	NC_042410	Li et al. (2019)
*A. sibirica*	Europe	NC_035067	Wu et al. (unbublished)
*A. ferreana*	Asia	NC_062889	Wang et al. (2022)
*A. fargesii*	Asia	NC_042775	Guo and Xu (2019)
*A. fabri*	Asia	NC_057314	Shao et al. (2020)
*A. chensiensis*	Asia	NC_041464	Zhao et al. (2019)
*A. chensiensis*	Asia	MH706706	Su et al. (2019)
*A. chensiensis*	Asia	MH047653	Liu et al. (2018)
*A. chensiensis*	Asia	MH510244	Li et al. (2018)

**TABLE 2:** Primer sequences, repeat motifs, approximate fragment lengths, melting temperatures, GC content, number of observed alleles and polymorphic information content (PIC) in *Abies grandis* for 17 introduced polymorphic primer pairs.

Primer	Primer sequence (5′‐3′)	Repeat motif	Fragment length	Tm (°C)	GC content (%)	Observed alleles	PIC
*AGcp01*‐F	TTCCAACCCCAAGTCTGGTC	C(12)	202	59.52	55	4	0.200
*AGcp01*‐R	GGATCGGAAATTGCATAAGCCTC			60.06	48		
*AGcp02‐*F	AAGTAGCTCCTGGATGGGGA	A(18)	309	59.66	55	4	0.439
*AGcp02*‐R	TTTCAACAAGTCGCACACCC			59.26	50		
*AGcp03*‐F	TCACACTGCTTTTCGGAGGG	T(17)	124	60.25	55	2	0.699
*AGcp03*‐F	TCGTGAAGCGAGAAAGGTGT			59.61	50		
*AGcp04*‐F	TCATTGGGTTCYTTGGRCCTT	G(12)	214	60.13	48	3	0.207
*AGcp04*‐R	TTGCCTCTCCTGATGGTTGG			59.67	55		
*AGcp05*‐F	TCGATCCATTTCCACCGGTAT	T(15)	154	58.96	48	5	0.419
*AGcp05*‐R	TCCGATCTGAATTACGGAAACCT			59.55	43		
*AGcp06*‐F	TGGACCATGAAAATGAAAGAATGGA	A(16)	205	59.22	36	5	0.634
*AGcp06*‐R	TGGGTAAGTCTTAGGACCCG			58.14	55		
*AGcp09*‐F	ACCTCAGCTATGTCCCTCGT	A(11)	201	60.03	55	3	0.338
*AGcp09*‐R	CGATCGGTCGCCAGGATAAA			59.97	55		
*AGcp11*‐F	GGGAAGAAAGAACATTTGGAAAACA	T(16)	205	58.65	36	6	0.674
*AGcp11*‐R	ACGTAATCTCCGGGATCCTTATT			58.84	43		
*AGcp15*‐F	ACCATTCAACCATACCCGCA	AT(7)	173	59.67	50	3	0.223
*AGcp15*‐R	AATGAAGGTGCTCAAGGGAGG			59.99	52		
*AGcp18*‐F	TTGGTACGGCACTTGAGAGA	T(17)	169	58.67	50	7	0.510
*AGcp18*‐R	AGTGACATCAATAACTGGTCCAA			57.76	39		
*AGcp19*‐F	CATGCCAACCACTCAACTCAC	A(13)T(5)	230	59.73	52	3	0.201
*AGcp19*‐R	TGACGTGGTGGAAGTCATCAG			60	52		
*AGcp20*‐F	GTGTTCCTCTATCCGTGGAGT	T(20)	207	58.9	52	8	0.569
*AGcp20*‐R	GAGGCGTACATCTCTTCTGGT			59.25	52		
*AGcp21*‐F	GCTTACCCTACATGGTCGAGA	A(19)	123	58.97	52	8	0.803
*AGcp21*‐R	TTCCTGGTATTGTCCAAGAATAGT			57.36	38		
*AGcp22*‐F	CTGCTGGATGCAGAGGAACT	T(14)	161	59.75	55	3	0.493
*AGcp22*‐R	TCCGATGGATTGTTACTGTGTATTG			59.18	40		
*AGcp23*‐F	TGGATTCGGTCCATTGATTGC	T(9)‐A‐C(9)T(7)	178	58.98	48	8	0.780
*AGcp23*‐R	CCATATTAGTTGACACRAKMTTTCA			57.23	36		
*AGcp24*‐F	GACCGATCATTGCGGGTACA	T(16)	334	60.18	55	6	0.510
*AGcp24*‐R	ATCCTCATGGAGGTGGGGAA			59.95	55		
*AGcp25*‐F	ACCCTTTTCCGAGGGGTAGT	T(23)	351	60.18	55	10	0.773
*AGcp25*‐R	ACCTCATACGGCTCCTCCTT			60.03	55		

### 2.2 Material

Sample material from *A. grandis* originates from a sample collection in the German IUFRO provenance trials in Lower Saxony, Hesse and North Rhine Westphalian. In total, 96 *A. grandis* individuals from all seed provenance regions (Rau et al., 2008) in the natural distribution range were included (Table A3). Sample material for other conifer species stem eighter from a sample collection in the Forest Botanical Garden of the University of Göttingen and the Arboretum in Hørsholm of the University of Copenhagen or were stored DNA samples of projects by the Department of Forest Genetics and tree breeding of the University of Göttingen or by ISOGEN GmbH & Co. KG (for details see Table A4). Also, 96 samples including 12 *Abies* spp., 2 *Cederus* spp., *Larix decidua*, *Picea abies*, *Pinus sylvestris*, *Pseudotsuga menziesii* and *Taxus baccata* were used in the transferability tests.

### 2.3 DNA extraction

From all fresh sample, genomic DNA was extracted from mature needle tissue. We used approximately 50 mg fresh or 25 mg dried materials cut into small pieces, frozen in liquid nitrogen and ground in an MM300 ball mill (Retsch, Haan, Germany) for 2 min at 30 Hz. For the extraction, we used the DNeasyTM 96 Plant Kit (Qiagen, Hilden, Germany) according to the manufacturer's protocol. Stored DNA samples were previously extracted in the same way. For *P. abies*, 5 mL of 26% polyvinylpyrrolidone solution was added to the 90.5 mL lysis buffer during the extraction.

### 2.4 Primer pair amplification and scoring

Primer testing was done performing PCR in a Biometra TProfessional thermocycler (Analytik Jena, Jena, Germany) with a touchdown protocol, consisting of 15 minutes initial denaturation (95°C), followed by 10 cycles of 60 s of denaturation (94°C), 60 s annealing (60°C; Δ‐1°C each cycle) and 60 s extension (72°C). Subsequently, another 25 cycles were performed, using the same temperatures and times, but maintaining 50°C in the annealing step. The protocol was finalized by 20 minutes of extension (72°C). Each volume (15 μL) contained about 2 ng of sample DNA, 1.05 pmol Tris–HCl and 0.263 pmol (NH4)2SO4, 37.5 pmol MgCl2, 2.5 pmol of each dNTP and 1U HOT FIREPol® Taq polymerase (Solis BioDyne, Tartu, Estonia). Polymorphic primer pairs were multiplexed with up to four primer pairs. Each sample volume contained 2.5 pmol M13 Primer (fluorescent dye labelled), 0.5 pmol forward and 1.25 pmol reverse primer of each tested primer pair in the respective multiplex. Each forward primer had an M13‐tail addition to the designed sequence.

The fragment sizes of the PCR products were determined using an ABI Genetic Analyzer 3130xl (Applied Biosystems, Foster City, CA, United States) and the accompanying Genemapper software v3.7 (Applied Biosystems, Foster City, CA, United States) (Figure A2).

Initial tests for successful amplification, fragment polymorphisms and good delimitation between fragment sizes were assessed using 16 samples of four distinct seed provenances. Subsequently, the polymorphic and well‐distinguishable primer pairs were tested in an enlarged data set of 96 *A. grandis* samples across the distribution range to estimate the degree of polymorphism (Table A3). Seed provenance regions follow the classification according to Rau et al. (2008). To test the reproducibility and verify the scoring of the data, an independent PCR, according to the protocol described above, was performed on these 96 samples. Additionally, we tested all polymorphic primer pairs in a sample set composed of 12 different related *Abies* and seven other conifer species (Table 3/Table A4).

**TABLE 3:** Number of alleles for each of the polymorphic *AGcp* primer pairs in various conifer species.

	*N*	*AGcp*‐No.																
		01	02	03	04	05	06	09	11	15	18	19	20	21	22	23	24	25
*Abies alba*	*8*	2	4	4	2	2	3	2	2	1	5	4	2	3	2	5	2	5
*Abies balsamea*	*2*	1	1	1	1	1	1	1	1	1	2	1	1	1	1	1	1	1
*Abies borisii regis*	*8*	1	3	5	2	3	3	3	2	1	5	4	3	4	4	3	4	4
*Abies cephalonica*	*4*	1	2	3	2	2	2	3	1	1	3	2	2	2	2	2	3	3
*Abies concolor*	*3*	2	2	2	2	2	2	1	2	1	1	2	2	2	2	2	1	2
*Abies fargesii*	*3*	1	2	3	1	2	3	1	1	3	2	3	1	3	3	3	2	3
*Abies holophylla*	*5*	3	2	3	3	2	3	1	1	1	2	2	2	4	3	2	1	2
*Abies homolepis*	*5*	1	3	3	2	3	2	1	1	1	3	3	1	3	2	3	3	4
*Abies koreana*	*3*	2	1	2	2	1	2	1	1	1	1	2	1	1	1	2	1	3
*Abies magnifica*	*2*	1	1	2	1	2	2	1	2	1	2	2	2	2	2	2	1	2
*Abies nordmanniana*	*5*	1	2	4	2	2	2	3	1	1	4	3	2	4	2	3	3	3
*Abies pinsapo*	*4*	1	1	2	1	2	3	1	1	1	1	3	2	2	2	3	1	3
*Cedrus atlantica*	*4*	1	1	2	2	0	1	1	1	1	0	0	0	0	0	0	2	3
*Cedrus libani*	*4*	2	1	1	1	0	2	1	1	1	0	0	0	0	0	0	1	3
*Larix decidua*	*8*	3	1	3	3	1	1	1	1	0	0	0	0	1	1	0	1	1
*Picea abies*	*8*	2	1	1	2	0	1	0	1	0	0	0	0	1	1	0	2	2
*Pinus sylvestris*	*8*	1	1	1	1	0	1	1	1	0	0	0	0	0	1	0	1	2
*Pseudotsuga menziesii*	*8*	3	1	1	4	1	0	1	1	0	0	0	0	1	1	0	2	3
*Taxus baccata*	*4*	1	1	1	0	0	0	0	0	0	0	0	0	0	0	0	1	0

*Note*: Color code highlights whether loci are polymorphic (green), did successfully amplify (yellow) or did not sufficiently amplify (no color) in the tested species. The number of individuals used per species (*N*) is indicated in italic.

### 2.5 Haplotype network construction and PCI calculation

A network of the detected haplotype in the 96 samples of *A. grandis* was constructed with NETWORK 10.2.0.0 (fluxus‐engineering.com) using the median‐joining algorithm (Bandelt et al., 1999). Visualization was performed in Gephi 0.10 (Bastian et al., 2009) using Yifan Hu's proportional layout algorithm (Hu, 2005) followed by manual adjusting for readability, avoiding overlaps and accounting for mutation steps, final post‐processing of the figure was done in Inkscape 1.1 (Inkscape Project, 2021) including inserting pie charts of occurrence frequency in different provenances generated in R 4.2.2 (R Core Team, 2022). Polymorphic information content (PCI) was calculated using the PopGenUtils 0.1.8 R package (Tourvas, 2021).

## 3 RESULTS

We developed 17 highly polymorphic primer pairs, which exhibited up to 10 different alleles in a comparatively small data set of 96 individuals (Table 2). All developed primer pairs amplify fragment sizes between 70 and 450 bp, contain between 36 and 63% Guanine and Adenine (mean ~ 50.1%) and are characterized by low chances of forming hair‐pin structures (Table A5). Melting temperatures ranged between 57°C and 61°C for all primers, with less than 2°C difference between primers of the same pair. The majority of the promising SSR candidates (repetitive motifs, polymorphic between different genomes) amplify single base pair repetitive motifs. We selected two double base pair motives, as well. However, only one of those (*AGcp15*) occur to be polymorphic in the primer tests. Despite the mostly single base pair repeats reproducibility was >99% over all primer in the 96 *A. grandis* samples in the comparison of the two independently performed PCRs and scorings.

All polymorphic primers amplified in all of the 12 tested *Abies* species (Table 3). Despite the small sample sizes (2 ≤ *n* ≤ 8) for each species apart from *A. grandis*, we observed polymorphisms in the majority of successfully amplified primer pairs across all *Abies* species. For conifer genera apart from *Abies*, amplification was often not successful for single primer pairs. However, we observed polymorphic loci in some genera, such as *Cedrus*, *Larix*, *Picea*, *Pinus* and *Pseudotsuga*. Only within the *Taxus* genus none of the primer pairs occurred to be polymorphic.

**FIGURE A1:**
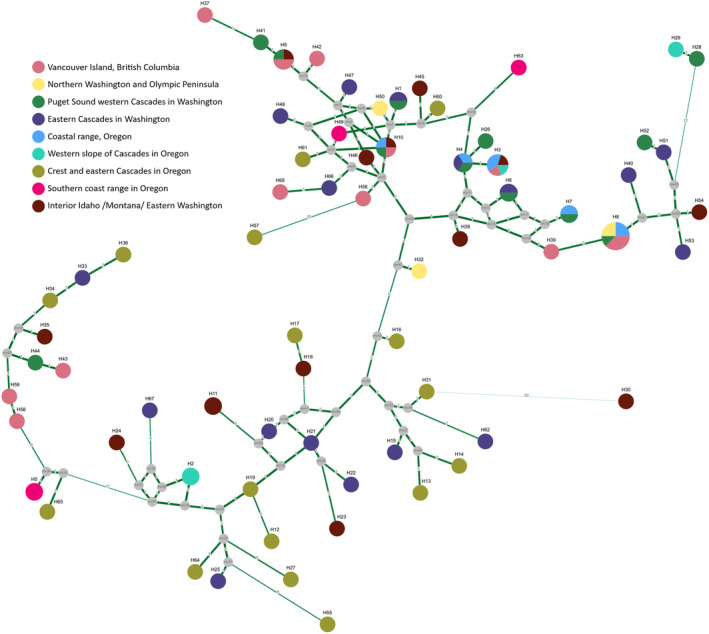
Median vector haplotype network of the detected 68 variants at 11 cpSSRs in 96 samples of Grand Fir (*Abies grandis*) across the distribution range. Haplotypes are named numerically with the prefix ‘H’ and coloured according to the origin of the sample/s containing respective haplotype. Median vectors are displayed as grey circles. Mutation steps are indicated as numbers at and thickness of the connecting edges. Construction of the network was performed with NETWORK 10.2.0.0 (fluxus‐engineering.com) using the median‐joining algorithm (Bandelt et al., 1999).

**FIGURE A2:**
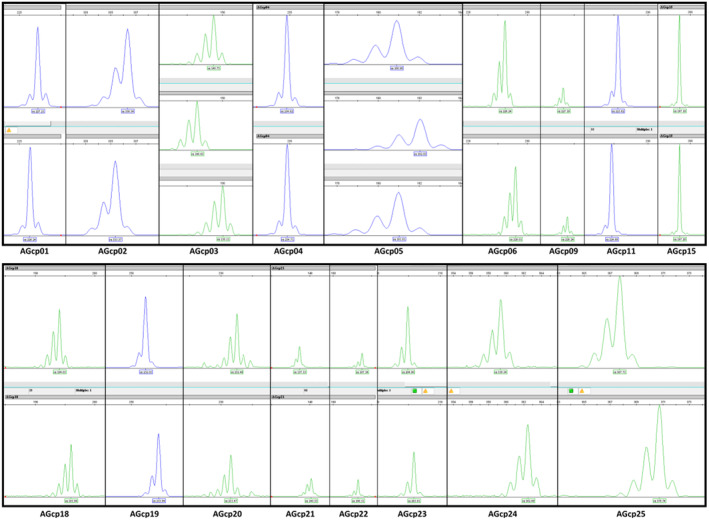
Representative peaks of different alleles for each of the introduced *Abies grandis* SSR markers as displayed in the ABI Genetic Analyzer 3130xl.

The haplotype network (Figure A1) shows a group of more closely related haplotypes, including those haplotypes that occur multiple times and in different regions of origin. Within the network, no clear separation of regions based on haplotypes can be observed.

## 4 DISCUSSION

The introduced cpSSR set for *A. grandis* is remarkably polymorphic also in other conifers especially *Abies* spp. Using 17 novel cpSSRs, we were able to define 68 different haplotypes from a sample set of only 96 *A. grandis* individuals. Previous studies already provided evidence for high diversity in *Abies* chloroplast haplotypes (e.g. Parducci et al., 2001; Vendramin et al., 1999), even using a limited amount of cpSSR markers. However, the introduced marker set of 17 cpSSR markers provides a valuable addition to the available marker set. Using additional genetic markers enables higher resolution of the genetic diversity, especially in geographically widespread studies. All of those novel cpSSR markers are also well suited for multiplex approaches and might thus contribute to a time and labour effective workflow. Finally, the transferability of some markers to additional species provides a functional tool for chloroplast haplotype definition in species of other genera, such as *Pseudotsuga menziesii*, or *Picea abies*. Certainly, the broad coverage of the distribution range contributed to this high diversity. Moreover, with up to 10 different alleles per primer pair, it is very likely to find even more haplotypes in larger datasets. Together with the option to use the marker set in several different *Abies* species, its highly polymorphic character makes it a well‐suited toolkit for future studies across a wide range of *Abies* species. The low number of polymorphic cpSSRs in *A. balsamea* might be due to the fact that only two seeds from the same seed source (Canda, Quebec) were analysed. The primer pairs *AGcp*01, *AGcp*02, *AGcp*03 and *AGcp*24 amplified in all tested conifer species and additional primer pairs amplified also in some of the non‐*Abies* species from different conifer genera. However, for very few primer pair‐species combinations, the PCR protocol might be not ideal and could be optimized for usage. The majority of the peaks were very clearly recognizable and easy to delimit. Despite their small sample sizes (*n* ≤ 8), some of the primer pairs, such as *AGcp*01, *AGcp*04, *AGcp*24 and *AGcp*25, even had various alleles in some tested non‐*Abies* species. Thus, it is likely, that some of the loci exhibit additional alleles in the tested species since our test plate contained only a small sample count of each species. Therefore, primer pairs, which were successfully amplified in the tested species, are promising candidates for those genera.

Despite the predominantly single base pair repeat motifs, the introduced cpSSR set exhibited reliable peaks, which could easily be kept apart and showed >99% reproducibility between repetitions. We chose highly conservative regions to design both, the forward and reverse primers on the upstream and downstream strands. No primers were developed for polymorphic regions in the alignment. SSR primer pairs from such regions would probably not result in reliable results since every possible primer pair would harbour the risk of several SNPs within the binding region. Such polymorphisms could compromise the annealing of the primers, possibly leading to non‐amplification of the targeted region in some samples. To examine such high polymorphic gene regions, SNP screening would probably be more promising due to the high abundance of SNPs in this region. However, some of the introduced primer pairs are positioned within gene sequences (Table S1) and, therefore, might be correlated to adaptive traits. Primer pairs excluded after the first test with 16 *A. grandis* samples could still be of interest in other *Abies* spp. Comprehensive information about all remaining primer pairs is reported in Table S2.

## 5 CONCLUSION

We have developed a reliable and user‐friendly cpSSR marker set for the relatively sparsely investigated species *A. grandis*. In a relatively small sample set, the marker set has already shown remarkable diversity. Further studies building on this marker set will contribute to the understanding of genetic patterns within the natural distribution range of *A. grandis*. Additionally, we demonstrated the usability of the set in other *Abies* spp. and conifer genera, improving upon the mostly RFLP‐based marker systems available for the chloroplast genome in conifers.

## AUTHOR CONTRIBUTIONS

**Jeremias Götz:** Data curation (equal); formal analysis (equal); investigation (lead); writing – original draft (lead); writing – review and editing (equal). **Ludger Leinemann:** Funding acquisition (equal); resources (equal); supervision (equal); writing – review and editing (equal). **Oliver Gailing:** Funding acquisition (equal); resources (equal); supervision (equal); writing – review and editing (equal). **André Hardtke:** Resources (equal); writing – review and editing (equal). **Oliver Caré:** Conceptualization (lead); data curation (equal); formal analysis (equal); investigation (supporting); methodology (lead); resources (equal); visualization (lead); writing – review and editing (equal).

## FUNDING INFORMATION

This research was funded by the German Federal Ministry of Food and Agriculture (BMEL) represented by the Fachagentur Nachwachsende Rohstoffe e. V. (FNR) grant number FKZ 2220NR313A. We acknowledge support by the Open Access Publication Funds of the Göttingen University.

## CONFLICT OF INTEREST STATEMENT

The authors declare no competing interests that are relevant to the content of this article.

## Supporting information


Tables S1–S2

## Data Availability

Genotype data are available as a Tables S1 and S2 with the online version of the current paper.

## Appendix

**TABLE A1:** Position of 17 polymorphic *AGcp* SSR amplicons, relative to *Abies* chloroplast genes.

Primerpair	Closest genes/proteins	Amplicon position, relative to genes
AGcp01	psbK	Overlapping amplicon, adjacent to 3′ end of gene sequence, overlapping
AGcp02	psbH	Overlapping amplicon, adjacent to 5′ end of gene sequence, overlapping
AGcp03	L16, S12	Inside gene sequence
AGcp04	rps12, psbZ	Inside rps12 sequence, adjacent to 3′ end of psbZ sequence, overlapping
AGcp05	rps12	Inside gene sequence
AGcp06	rps12, rsp15	Inside rps12 gene sequence, adjacent to 3′ end of rsp15 sequence, overlapping
AGcp07	rps12	Inside gene sequence
AGcp08	trnL‐CAA	Adjacent to 3′ end of gene, overlapping amplicon
AGcp09	trnK‐UUU	Inside gene sequence
AGcp10	trnK‐UUU	Adjacent to 5′ end of gene, non‐overlapping amplicon
AGcp11	psbI	Adjacent to 3′ end of gene, non‐overlapping amplicon
AGcp12	psbT, psbN	Amplicon between gene sequences, overlapping both sequences
AGcp13	rps12, petD	Inside both gene sequences
AGcp14	rps12, rlp14, rlp16	Inside rsp 12gene sequence, overlapping 5´end of rlp14 gene sequence and 3´end of rlp 16 gene sequence.
AGcp15	rps12, trnG‐UCC	Inside rsp 12gene sequence, adjacent to 5′ end of trnG‐UCC gene sequence, overlapping amplicon
AGcp16	rps12	Inside gene sequence
AGcp17	rps12	Inside gene sequence
AGcp18	rps12	Inside gene sequence
AGcp19	rps12, trnV‐GAC	Inside rsp12 gene sequence, amplicon adjacent to 5′ end of trnV‐GAC gene sequence, overlapping
AGcp20	‐	No genes nearby
AGcp21	‐	No genes nearby
AGcp22	trnI‐CAU	Adjacent to 5′ end of gene, non‐overlapping amplicon
AGcp23	ycf2	Adjacent to 5′ end of gene, non‐overlapping amplicon
AGcp24	rps12, rsp19, rpl2	inside rsp 12 gene sequence, amplicon adjacent to 5′ end of rsp19, and 3# end of rpl2 gene sequence, overlapping both sequences
AGcp25	rps12, ycf3	Inside both gene sequences

**TABLE A2:** Information about the initially non‐polymorphic chloroplast SSR markers, developed for *Abies grandis*.

Primer	Primer sequence (5′‐3′)	Pepeat motif	Fragment length	Tm (°C)	GC content (%)
AGcp07‐F	TACCATCCCCATCAGAACGA	T(13)	259	57.83	50
AGcp07‐R	GACGATCATAGGTCGGGAGT			58.39	55
AGcp08‐F	ATCGATATCAATACTCCAATACGCT	TA(8)	149	57.9	36
AGcp08‐R	TTTGAGTCTCGCGTGTCTACC			60.07	52
AGcp10‐F	GTYGCACGTTGCTTTCTACC	T(5)	70	59.84	55
AGcp10‐R	TGATTCGAGATCAGSAGGGAG			59.31	52
AGcp12‐F	TTCTTCCGAGAACCACCCAA	C(4)	177	58.87	50
AGcp12‐R	TGGGCAACCCTCTGAACAAC			60.47	55
AGcp13‐F	ATGGTCCTCGGATTTCTCCT	A(5)	241	57.82	50
AGcp13‐R	TGACTGATGCTTTTCCACCCA			59.86	48
AGcp14‐F	CGGGCTCCACTGTTATCCG	A(14)	436	60.23	63
AGcp14‐R	GCAGATAGAAGCCGGACGAA			59.9	55
AGcp16‐F	TGAAGCGAATGGAGACAAGTTA	C(8)	124	57.73	41
AGcp16‐R	TCCCTACCCTCTCGTGTCAA			59.59	55
AGcp17‐F	TGGAGACTGGGAATTRACGG	T(16)	232	59.39	55
AGcp17‐R	GCACGGATAGAAGTGGGTGA			59.46	55

**TABLE A3:** Origin of the genetic material of the 96 *Abies grandis* samples from the different German IUFRO trials.

Origin	Trial	Sample size
Vancouver Island, Brit. Kolumbien	IUFRO Lauterberg, Germany	16
Olympic Peninsula, Washington	IUFRO Lauterberg, Germany	4
Puget Sound, Washington	IUFRO Lauterberg, Germany	12
Central Washington	IUFRO Hochstift, Germany	12
Coastal mountain range, Oregon	IUFRO Lauterberg, Germany	12
Western Oregon, Main land	IUFRO Lauterberg, Germany	4
Southern coast, Oregon	IUFRO Lauterberg, Germany	4
Central Oregon	IUFRO Lauterberg, Germany	16
Idaho	IUFRO Langen, Germany	16

**TABLE A4:** Origin of the genetic material of the 96 samples of other *Abies* spp. and other conifers.

Species	Sample origin	Provenance	Sample size	
*Abies alba*	Arboretum Copenhagen	Lapos, Romania	1	8
	ISOGEN GmbH& Co.KG	Lower Saxony, Germany	7	
*Abies balsamea*	Arboretum Copenhagen	Quebec, Canada	2	2
*Abies borisii regis*	Forest Botanical Garden Göttingen	x	2	8
	ISOGEN GmbH & Co.KG	Slavjanka Mountanis, Greece/Bulgaria	6	
*Abies cephalonica*	Forest Botanical Garden Göttingen	x	4	4
*Abies concolor*	Arboretum Copenhagen	New Mexico, Santa Fee, Blue Bird Mesa, USA	1	3
	Forest Botanical Garden Göttingen	x	2	
*Abies fargesii*	Arboretum Copenhagen	Gansu (2) and Sichuan (1), China	3	3
*Abies holophylla*	Arboretum Copenhagen	Gangwon, South Korea (3) and Forest in Denmark (2)	5	5
*Abies homolepis*	Arboretum Copenhagen	Shizouka, Japan	2	5
	Forest Botanical Garden Göttingen	x	3	
*Abies koreana*	Arboretum Copenhagen	Jeju Iland, South Korea	3	3
*Abies magnifica*	Forest Botanical Garden Göttingen	x	2	2
*Abies nordmanniana*	Arboretum Copenhagen	Sups. equi‐trojani, Denmark	1	5
	ISOGEN GmbH & Co.KG	Sups. bornmuelleriana Kökez, Bolu, Turkey (2), sups. ambrolauri Caucasus, Georgia (2)	4	
*Abies pinsapo*	Forest Botanical Garden Göttingen	x	1	4
	Arboretum Copenhagen	Cadiz, Spain (1) and Rif Mts., Marocco (2)	3	
*Cedrus atlantica*	ISOGEN GmbH & Co.KG	North Rhine‐Westphalia, Germany	4	4
*Cedrus libani*	ISOGEN GmbH & Co.KG	North Rhine‐Westphalia, Germany	4	4
*Larix decidua*	ISOGEN GmbH & Co.KG	Kusel, Rhineland‐Palatinate, Germany	8	8
*Picea abies*	Department of Forest Genetics and Forest Tree breeding	Low‐elevation phenotype, Harz Mts.; Germany	4	8
		High elevation phenotype, Thuringia, Germany	4	
*Pinus sylvestris*	ISOGEN GmbH & Co.KG	Thuringia, Germany	8	8
*Pseudotsuga menziesii*	ISOGEN GmbH & Co.KG	var. menziesii, North Rhine‐Westphalia, Germany	4	8
	Department of Forest Genetics and Forest Tree breeding	var. glauca, IUFRO trial Germany	4	8
*Taxus baccata*	Department of Forest Genetics and Forest Tree breeding	Bavaria, Germany	4	

*Note*: Sample origin refers to the place of sampling or the institution where samples were collected/stored and provenance for known geographic origin of the material.

**TABLE A5:** Self complementarity scores of primers according to NCBI Primer blast server application (Ye et al., 2012).

Primer	Self complementarity	Self 3′ complementarity
*AGcp01*‐F	3	1
*AGcp01*‐R	4	1
*AGcp02‐*F	6	0
*AGcp02*‐R	3	0
*AGcp03*‐F	3	0
*AGcp03*‐F	3	0
*AGcp04*‐F	4	0
*AGcp04*‐R	2	0
*AGcp05*‐F	6	2
*AGcp05*‐R	6	0
*AGcp06*‐F	5	0
*AGcp06*‐R	7	2
*AGcp09*‐F	4	0
*AGcp09*‐R	6	0
*AGcp11*‐F	4	0
*AGcp11*‐R	6	1
*AGcp15*‐F	3	0
*AGcp15*‐R	3	0
*AGcp18*‐F	4	0
*AGcp18*‐R	5	3
*AGcp19*‐F	4	0
*AGcp19*‐R	4	1
*AGcp20*‐F	4	2
*AGcp20*‐R	6	0
*AGcp21*‐F	4	2
*AGcp21*‐R	5	2
*AGcp22*‐F	6	1
*AGcp22*‐R	4	1
*AGcp23*‐F	4	2
*AGcp23*‐R	4	2
*AGcp24*‐F	4	2
*AGcp24*‐R	6	0
*AGcp25*‐F	4	1
*AGcp25*‐R	2	0
